# Phosphate in Physiological and Pathological Mineralization: Important yet Often Unheeded

**DOI:** 10.1002/mco2.70298

**Published:** 2025-07-13

**Authors:** Wen Qin, San‐yang Yu, Jia‐lu Gao, Jian‐fei Yan, Qian‐qian Wan, Shuai‐lin Jia, Franklin Tay, Kai Jiao, Lina Niu

**Affiliations:** ^1^ State Key Laboratory of Oral and Maxillofacial Reconstruction and Regeneration, National Clinical Research Center for Oral Diseases & Shaanxi Key Laboratory of Stomatology, Department of Prosthodontics, School of Stomatology The Fourth Military Medical University Xi'an Shaanxi PR China; ^2^ School and Hospital of Stomatology China Medical University Shenyang Liaoning PR China; ^3^ Department of Stomatology, Tangdu Hospital, State Key Laboratory of Oral and Maxillofacial Reconstruction and Regeneration, National Clinical Research Center for Oral Diseases, Shaanxi Key Laboratory of Stomatology, School of Stomatology The Fourth Military Medical University Xi'an Shaanxi PR China; ^4^ State Key Laboratory of Oral & Maxillofacial Reconstruction and Regeneration, National Clinical Research Center for Oral Diseases, Shaanxi Clinical Research Center for Oral Diseases, Department of Oral and Maxillofacial Surgery, School of Stomatology The Fourth Military Medical University Xi'an Shaanxi PR China; ^5^ The Third Affiliated Hospital of Xinxiang Medical College Xinxiang Henan PR China; ^6^ The Dental College of Georgia Augusta University Augusta Georgia USA

**Keywords:** pathological mineralization, phosphate homeostasis, phosphate metabolism disorders, physiological mineralization, therapeutic strategies

## Abstract

Phosphate is an important element in biological processes, particularly in the formation and metabolism of mineralized tissues such as bones and teeth. The imbalance of phosphate is also closely related with pathological mineralization. Restoring the phosphate homeostasis is an attractive target to treat diseases related with pathological mineralization. However, the inherent consistency of phosphate's role in both physiological and pathological mineralization has been overlooked in previous investigations. This review highlights the multifaceted role of phosphate as a building block, and as a signaling molecule that regulates the activity of mineralizing cells in both physiological and pathological mineralization. This direct and indirect role of phosphate acts as a bridge between physiological and pathological mineralization. The review also discusses the genetic mutations associated with phosphate‐related mineralization disorders, emphasizing the need for further genetic and molecular research to uncover additional factors and mechanisms. Future research directions proposed include enhancing our understanding of phosphate sensing and regulation mechanisms, investigating new therapeutic agents, and developing reliable biomarkers for early diagnosis and treatment of phosphate‐related mineralization disorders. By advancing our knowledge in these areas, we can improve the prevention, diagnosis, and treatment of phosphate‐related mineralization disorders to enhance patient outcomes and their quality of life.

## Graphical abstract

Phosphate serves as a building block for physiological mineralization, and as a signaling molecule that regulates the activity of mineralizing cells. The disturbance in these processes could induce a series of pathological mineralization, with abnormal mineralization of hard tissues and ectopic mineralization of soft tissues being the most representative.

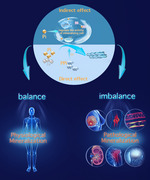

## 1 Introduction

Biomineralization is a precisely controlled process by which living organisms produce inorganic minerals through biochemical processes under biological control [1, 2, 3]. It is a common and significant phenomenon in nature. During long‐term evolution, the capacity of biomineralization has consistently maintained, from primitive eukaryotes approximately 810 million years ago to vertebrates including humans [4, 5, 6]. Biominerals are essential for growth, structural support, and protection in organisms [7]. In higher vertebrates, such as mammals, calcium phosphate is the predominant form of biominerals in bone and teeth [8]. The importance of calcium in biomineralization has been extensively reviewed in the previous studies [9, 10]. However, compared with calcium, phosphate has been frequently underestimated. Actually, some evidence suggests that phosphate may play a more important role in biomineralization process [11, 12].

The enrichment of phosphate and its regulation of mineralization are crucial for the mineralization process. For example, in the teeth formation of jaw fishes, the local accumulation of phosphate by cells is a prerequisite due to the low concentration of phosphate in marine. Additionally, phosphate also plays a more important role in the nucleation process at the initial mineralization stage [13, 14]. Another crucial aspect is that pyrophosphate (PPi) serves as the main mineralization inhibitor to maintain the nonmineralized stage of the soft tissue [15, 16]. Previous reviews have explored the regulatory effects of phosphate on mineralizing cells in physiological mineralization, and the changes of genes related to phosphate homeostasis in pathological mineralization [11, 17]. However, they overlook the fact that the inherent consistency of phosphate's role in both physiological and pathological mineralization.

Considering the essential role of phosphate in biomineralization, this review summarized the dual functions of phosphate in physiological and pathological mineralization. Phosphate directly participates in physiological mineralization as a building block for mineralization and indirectly acts as a signaling molecule that regulates the activity of mineralizing cells. The review also discusses pathological mineralization‐related diseases that are caused by disturbances of the dual regulatory functions of phosphate.

## 2 Regulation of Phosphate Homeostasis

Phosphate plays vital roles in various biological processes, including energy metabolism, protein regulation, metabolic pathways, genetic integrity, acid–base balance, and membrane structure. Phosphate homeostasis is tightly regulated through sensing mechanisms involving PiT‐1/PiT‐2 transporters, FGFR1, and calcium‐sensing receptors, which modulate hormones like FGF23 and PTH. Intestinal absorption and renal reabsorption/excretion maintain serum Pi levels. And the interorgan communication (bone–kidney–intestine–parathyroid axis) benefits phosphate homeostasis maintenance. This dynamic regulation is essential for biomineralization and overall physiological functions.

### 2.1 Distribution and Role of Phosphate in the Body

Total phosphate accounts for ∼0.6% of the body weight at birth, and ∼1% in adults, primarily existing in the form of organic and inorganic phosphates (Pi). The majority (80%–85%) of phosphate is located within the extracellular matrix (ECM) in the skeleton and teeth, in the form of apatite. The remaining phosphate (15%–20%) is found in soft tissues (as components of adenosine triphosphate [ATP], phospholipids, and phosphoproteins), and extracellular fluid (two‐thirds of it being organic and one‐third inorganic; Table 1) [18, 19]. Reaction involving phosphate esters and Pi is crucial in all biological processes. The stability of phosphate ester bonds makes them ideal for regulating information and energy transfer mechanisms [20]. Specialized enzymes lower the hydrolysis barrier, enabling precise control of biochemical reactions. Key examples include:

**TABLE 1:** The content of phosphate in different locations [18, 19].

	Organic phosphates	Inorganic phosphates
Category	DNA, RNA, phospholipids, and so forth	85% is free; 10% bond to protein; 5% is complexed with calcium or magnesium
Intracellular concentration	5–70 mmol/L	0.7 → 2 mmol/L
Extracellular concentration	Are not routinely measured	0.8 → 1.4 mmol/L (3–4.5 mg/dL)

(1) Intracellular energy transfer: ATP converts to adenosine diphosphate (ADP) and Pi, producing biochemical energy [21, 22].

(2) Regulation of protein function: phosphorylation serine, threonine, and tyrosine in proteins allow for versatile and reversible post‐translational modifications. This enables specific recognition and signal transmission [23, 24].

(3) Metabolism: phosphorylation is important in glycolysis, and oxidative phosphorylation, with glucose phosphorylation being the initial step in glycolysis [25].

(4) Maintenance of genetic material integrity: the stability of phosphate esters makes them suitable for storing genetic information in deoxyribonucleic acid (DNA) and ribonucleic acid (RNA) [26, 27].

(5) Regulation of acid/base balance: Blood phosphate exists as sodium dihydrogen phosphate (Na_2_H_2_PO_4_^−^) and sodium monohydrogen phosphate (Na_2_HPO_4_^2−^), acting as a buffer system to maintain pH [28].

(6) Formation of phospholipids: phosphate is a building block for the synthesis of phospholipids in all plasma and intracellular membranes [29, 30, 31, 32].

### 2.2 Mechanisms to Maintain Phosphate Homeostasis

#### 2.2.1 Phosphate Sensing

**FIGURE 1:**
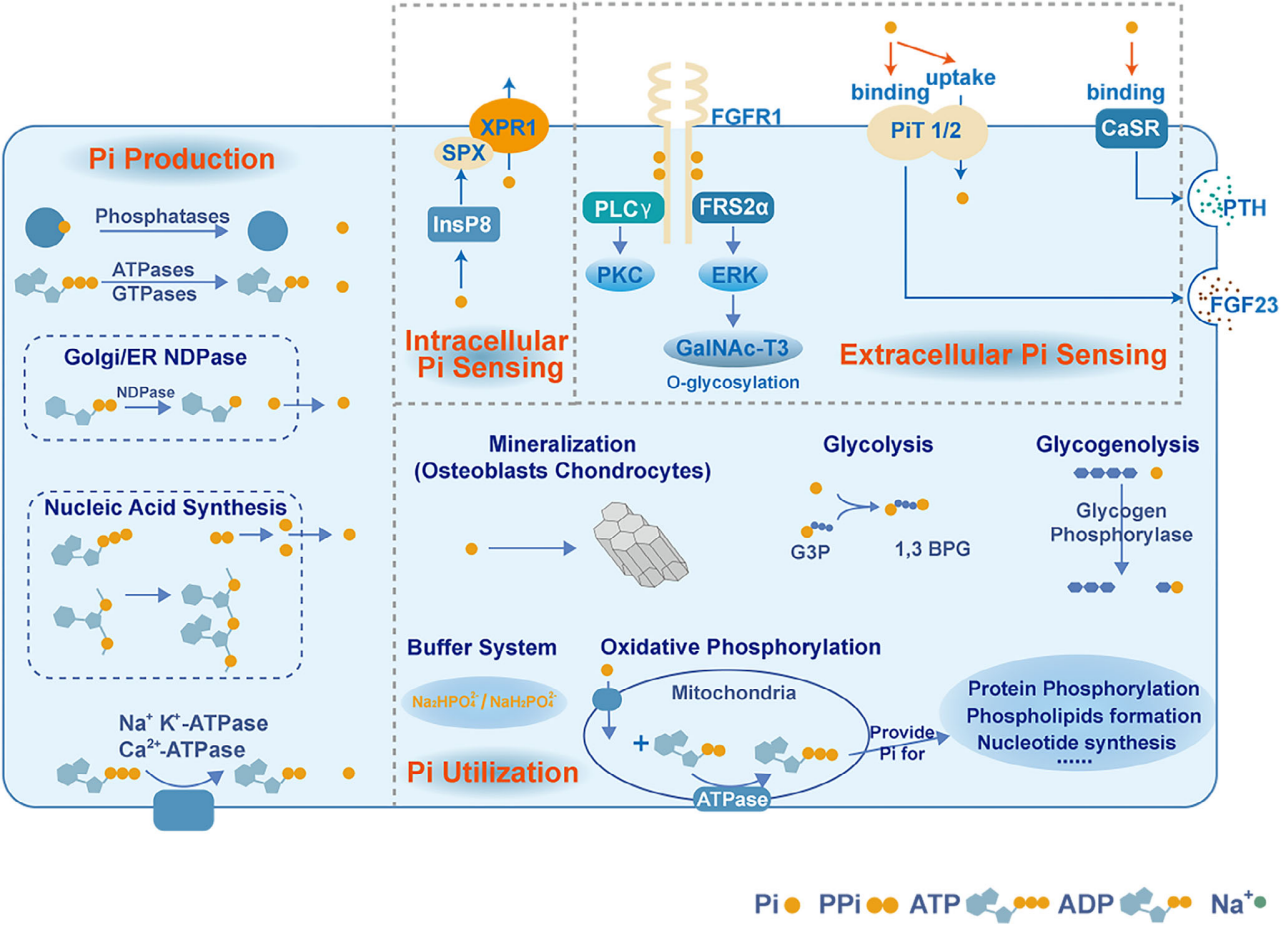
Metabolic process related to Pi production, Pi utilization, and Pi transport regulation. Pi production: Various cellular metabolic activities generate Pi. Intracellular Pi sensing: InsP8 senses intracellular Pi levels and binds to the SPX domain of XPR1, facilitating Pi efflux. Extracellular Pi sensing: Extracellular high Pi levels activate FGFR1, triggering the MEK/ERK signaling pathway to induce GALNT3 expression. PiT‐1/2 heterodimers detect fluctuations in extracellular phosphate levels. PiT‐2 in bone senses phosphate changes and induces FGF23 secretion under high phosphate. High Pi levels inhibit the calcium‐sensing receptor (CaSR) in parathyroid cells, leading to increased parathyroid hormone (PTH) secretion. Pi utilization: Pi is utilized in various cellular processes.

Phosphate sensing is the pivotal step in maintaining phosphate metabolism (Figure 1). Cell sense variations in extracellular phosphate concentrations and modulate the secretion of hormones including parathyroid hormone (PTH), fibroblast growth factor 23 (FGF23) in response to these changes, thereby maintaining systemic phosphate homeostasis [33, 34]. Both phosphate transporter‐1 (PiT‐1) and PiT‐2 detect fluctuations in extracellular phosphate levels by forming heterodimers, a process independent of their phosphate transport function [35]. PiT‐2 in bone senses changes in phosphate levels and induces the synthesis and secretion of FGF23 in response to elevated phosphate conditions [36]. Fibroblast growth factor receptor 1 (FGFR1) functions as a key phosphate sensor, playing a crucial role in FGF23 production and the regulation of serum phosphate levels [37]. Extracellular phosphate directly activates FGFR1, leading to the phosphorylation of fibroblast growth factor receptor substrate 2α (FRS2α). This activation stimulates the MEK/ERK pathway and induces the expression of polypeptide N‐acetylgalactosaminyltransferase 3 (GALNT3), an enzyme that post‐translationally modifies FGF23 [38]. Calcium‐sensitive receptors serve as phosphate sensors in parathyroid cells. Elevated phosphate levels inhibit the activity of these receptors by noncompetitive antagonism, resulting in a rapid increase in PTH secretion [39, 40]. Additionally, it is necessary to sense intracellular phosphate concentrations as phosphate is the basic component of phospholipids, ATP, and so on [41, 42]. And the relevant research is relatively less but has been gaining increasing attention. A recent study has reported that the concentration of inositol polyphosphate (InsP) varies with the intracellular Pi level, indicating a possibility for intracellular Pi sensing. InsP_8_, and inositol hexakisphosphate kinases 1/2 have been reported as intracellular sensors. They can bind to SPX domain on the phosphate exporter xenotropic and polytropic retrovirus receptor 1 (XPR1), which serves as a Pi exporter in mammalian cells [43, 44, 45].

#### 2.2.2 Phosphate Transport and Storage

Phosphate required for mineralization is absorbed from food and enters the blood via active transcellular transport and passive paracellular transport in the small intestine [46, 47]. It is then transported to the bones and other tissues for accumulation [48]. Phosphate is freely filtered in the glomerulus and reabsorbed via active transport in the proximal tubule, or it is excreted [49, 50]. NaPi‐IIb is responsible for 90% of phosphate absorption in the small intestine, while PiT‐1 plays a compensatory role when NaPi‐IIb expression is reduced [51, 52]. PiT‐2 potentially regulates phosphate balance under low phosphate intake [53]. NaPi‐IIa interacts with Na^+^/H^+^ exchange regulatory factor‐1 (NHERF1) to form the NaPi‐IIa/NHERF1 complex, which affects renal phosphate reabsorption. Complex formation is controlled by PTH and FGF23 [54, 55, 56].

The concentration of serum Pi under physiological conditions is 0.8–1.4 mmol/L [18, 57]. Due to the regulatory effect of osteoblasts, the Pi concentration in the mineralized region is much higher than in the serum [58]. In the presence of elevated Pi levels, Pi accumulates on and around the endoplasmic reticulum in osteoblasts, resulting in the formation of localized Pi concentration area. Mitochondria absorb phosphate from these regions and, under the influence of ATP and mitochondrial oxidative phosphorylation, form amorphous mineral particles [59, 60, 61]. These particles are then transferred to extracellular vesicles through processes like diffusion and mitophagy, where they are secreted outside the cell to participate in mineralization [62, 63].

#### 2.2.3 Interorgan Communication for Phosphate Homeostasis Maintenance

**FIGURE 2:**
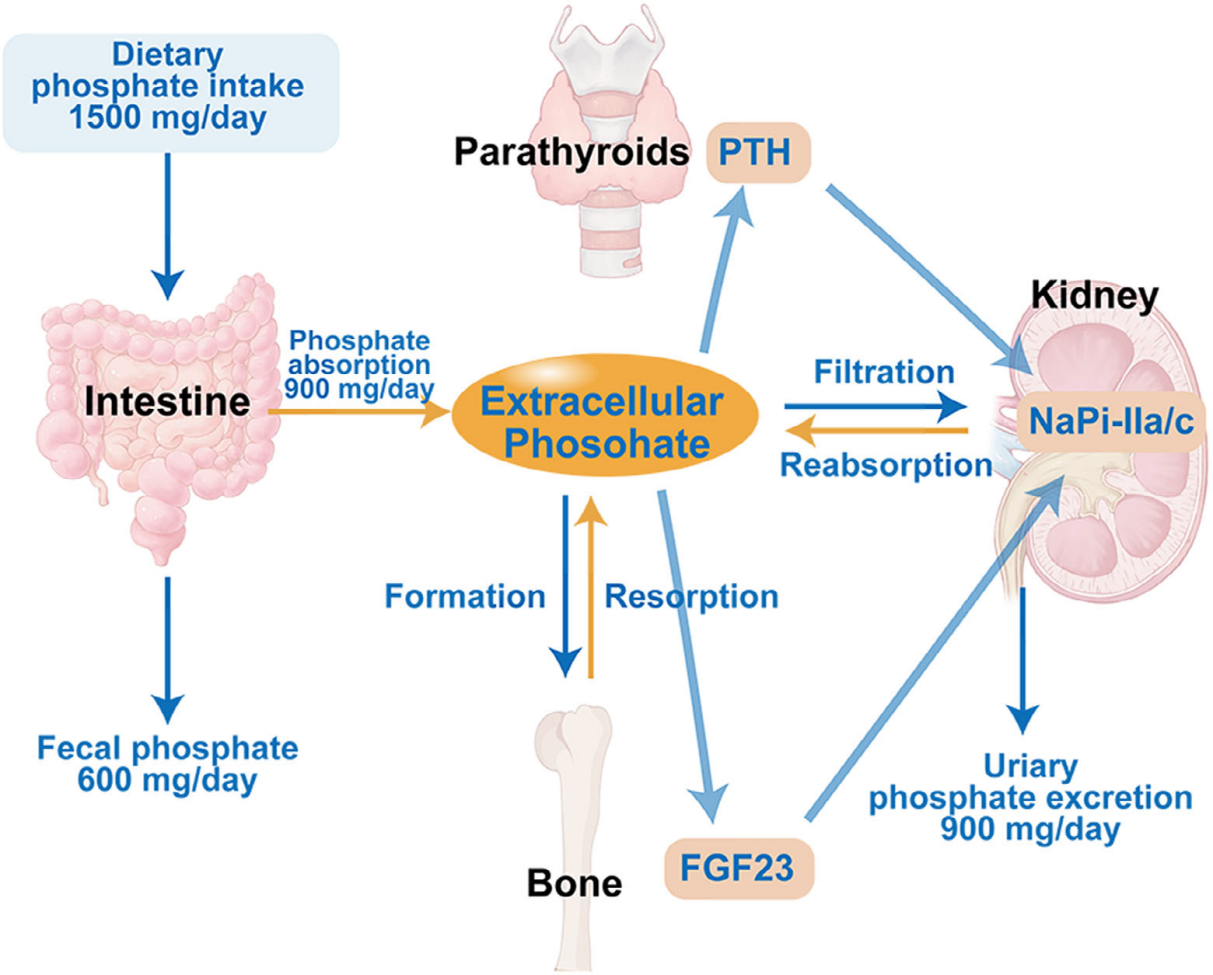
Regulatory mechanism of phosphate homeostasis in the human body. The maintenance of phosphate homeostasis depends on the interaction among the kidney, intestine, parathyroid glands, and bone. Adapted with permission from ref. [49]. Copyright 2016, International Society of Nephrology.

Dietary phosphates are absorbed by the intestines and transported into the bloodstream, accumulating in bone and other tissues. In plasma, phosphate undergoes filtration by the glomeruli and is either reabsorbed by the renal tubules or excreted [49]. These organs communicate with one another through the secretion of hormones to synergistically maintain phosphate balance (Figure 2) [64].

Bone regulates phosphate metabolism in an endocrine manner [65, 66]. The secretion of FGF23 by osteoblasts in bone is regulated by serum phosphate levels, PTH, and 1,25(OH)_2_D_3_ [67]. FGF23 specifically interacts with FGFR1 and FGFR4 in the kidneys, inhibiting the expression of NaPi‐IIa and NaPi‐IIc in the proximal renal tubules, leading to increased phosphate excretion in the urine [68, 69]. Additionally, FGF23 causes a decrease in 1,25(OH)_2_D_3_ synthesis and an increase in its degradation, thereby decreasing intestinal phosphate absorption [70, 71]. Furthermore, FGF23 engages in direct interactions with parathyroid cells via FGFRs, thereby reducing PTH secretion [72]. The PTH reduces the levels of NaPi‐IIa and NaPi‐IIc through the PTH receptor 1 in the proximal tubule cells of the kidney, thereby reducing the reabsorption of phosphate [73, 74]. PTH also promotes the expression of 1,25(OH)_2_D_3_ [75, 76]. 1,25(OH)_2_D_3_, the active form of vitamin D, is mainly synthesized in the kidneys. It promotes intestinal phosphate absorption by activating the transcellular pathway mediated by NaPi‐IIb [77, 78]. In addition, 1,25(OH)_2_D_3_ can upregulate the synthesis of FGF23 and inhibit its cleavage by inhibiting furin expression [79, 80]. The interaction between 1,25(OH)_2_D_3_ and FGF23 forms a negative feedback loop which helps to stabilize phosphate levels in the bloodstream [81]. Following alterations in dietary phosphate levels, PTH levels demonstrate rapid fluctuations within minutes [82]. Even in the absence of PTH and FGF23 receptor signaling, a high‐phosphate diet can rapidly downregulate renal Na^+^/Pi transporters [83].

Biomineralization is a dynamic and continuous process in which bones acquire the phosphate needed for mineralization from the serum. Phosphate directly contributes to mineralization or serves as a signaling molecule influencing multiple cells that regulate the mineralization process. The intricate phosphate‐sensing and regulatory mechanisms in the body are essential for maintaining phosphate homeostasis, which is critical for the biomineralization process [11].

## 3 The Dual Function of Phosphate in Physiological Mineralization

Phosphate plays both direct and indirect roles in physiological mineralization. Directly, the PPi/Pi ratio critically controls mineralization, as PPi inhibits while Pi promotes crystal formation. Indirectly, phosphate acts as a signaling molecule, modulating chondrocyte, osteoblast, and osteoclast activity‐promoting proliferation, differentiation, or apoptosis depending on concentration and cell type. Additionally, protein phosphorylation (e.g., osteopontin, amelogenin) fine‐tunes mineralization by stabilizing amorphous calcium phosphate (ACP) or promoting hydroxyapatite (HAP) formation, ensuring spatiotemporal control of biomineralization. These mechanisms collectively maintain balanced mineralization through dynamic phosphate regulation.

### 3.1 The Direct Effect: Phosphate Regulating Physiological Mineralization

In physiologically mineralized tissue like cartilage and woven bone, cells secrete matrix vesicles (MVs) containing calcium and phosphate into extracellular space and form carbonated apatite (CAP) within collagen fibrils. This process is known as MVs‐mediated mineralization [84, 85]. As the focal points for mineralization initiation, MVs require the local enrichment of calcium and phosphate to achieve sufficient concentration for mineralization [86]. The endoplasmic reticulum and mitochondria are considered major calcium reservoirs within cells [87, 88]. They participate in the formation of MVs and supply substantial calcium for ACP formation [89].

**FIGURE 3:**
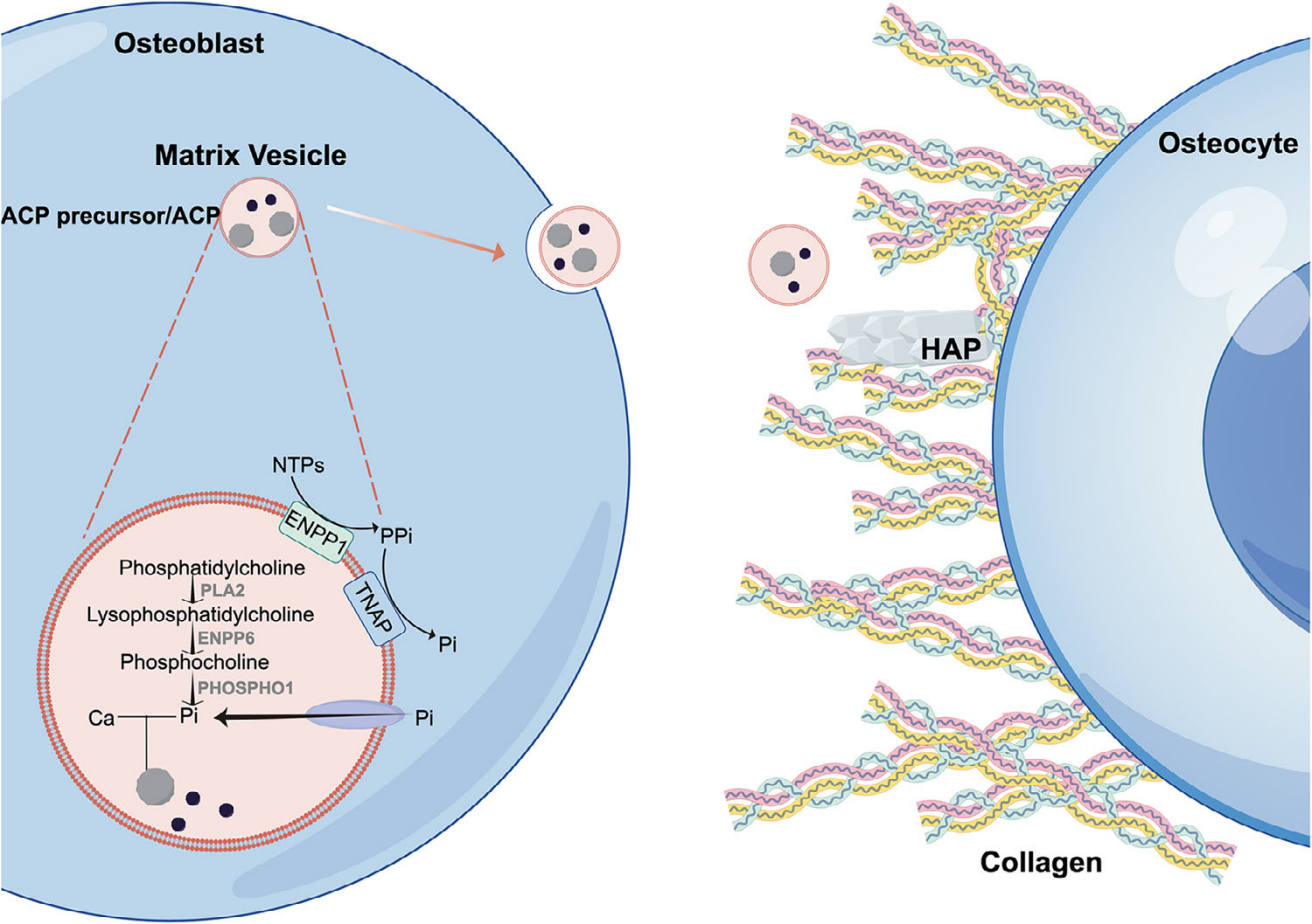
The direct effect of phosphate in physiological mineralization. Pi is enriched through several pathways inside the MVs and forms ACP with calcium. After secreted from osteoblasts, ACP in MVs is released onto the collagen and causes mineralization.

Phosphate, unlike calcium, lacks specialized cellular storage organelles and is mainly incorporated into organic phosphates such as phospholipids in the cell membrane [90]. Therefore, a sophisticated biological mechanism is necessary for MVs to achieve sufficient phosphate concentration in the MVs. Research has demonstrated that PHOSPHO1, tissue‐nonspecific alkaline phosphatase (TNAP), and ectonucleotide pyrophosphatase/phosphodiesterase 1 (ENPP1) in the MVs participate in the maintenance of phosphate balance within the MVs and in the extracellular environment (Figure 3) [86, 91, 92].

PHOSPHO1 is mainly responsible for Pi production within the MVs. This enzyme exhibits high affinity for phosphoethanolamine and phosphocholine, thereby serving as the substrate to convert into Pi [93]. However, the mechanisms of their synthesis are not fully understood. Notably, phosphatidylcholine constitutes the majority of the membrane of MVs, with the phosphatidylethanolamine being the second most abundant. Elucidating the transformation of phospholipids into phosphoethanolamine and phosphocholine represents an important avenue for future research [94, 95].

Pi can also be produced outside of the MVs and transported into MVs by proteins located on the membranes. ENPP1 facilitates the conversion of ATP to PPi, which is subsequently degraded into Pi by the action of TNAP [96, 97, 98]. This Pi is then imported into MVs by PiT‐1 transporter [97]. As is widely accepted, PPi exerts an inhibitory effect on mineralization [99, 100, 101]. Therefore, the precise control of the PPi/Pi ratio is essential for the mineralization process outside of the MVs [102, 103]. As indicated by previous studies [104, 105], ENPP1 is primarily expressed in mature osteoblasts and osteocytes, while TNAP is localized predominantly in osteoprogenitor cells and mature osteoblasts. This differential expression pattern may serve as a regulatory mechanism to prevent excess mineralization in the later phases of osteogenesis [104, 106].

When released from cells, MVs containing ACP deposit on collagen fibrils [107, 108, 109]. Nucleation and mineralization of ACP are initiated on the inner side of the MVs. This is because phosphatidylserine (PS) located in the inner leaflet of MVs membrane is capable of stabilizing prenucleation calcium phosphate complexes prior to ACP formation. The PS‐rich nanodomains create a local site for ACP nucleation. The promotion of stabilization and nucleation is driven by the highly negatively charged interface generated by the phosphate groups of PS [110, 111].

However, there is still controversy about whether the ACP or HAP crystals are released from MVs to collagen fibrils. Some researchers have proposed that ACP is transformed into octacalcium phosphate (OCP) and then into HAP crystals, which is mediated by the PPi/Pi ratio in MVs [112, 113]. Then HAP crystals in MVs are released into the ECM, which could explain the extrafibrillar mineralization of collagen. Others have suggested that ACP plays a more significant role in initiating collagen intrafibrillar mineralization and subsequent growth of HAP platelets, while crystals are less able to induce intrafibrillar mineralization [114, 115]. The exact mechanism of prevent the transformation of ACP into crystals in MVs is not fully understood. It is hypothesized that the PPi/Pi ratio might also play a crucial role in this process, which needs further investigation [84, 116].

### 3.2 The Indirect Effect: Phosphate Regulating Mineralizing Cells

**FIGURE 4:**
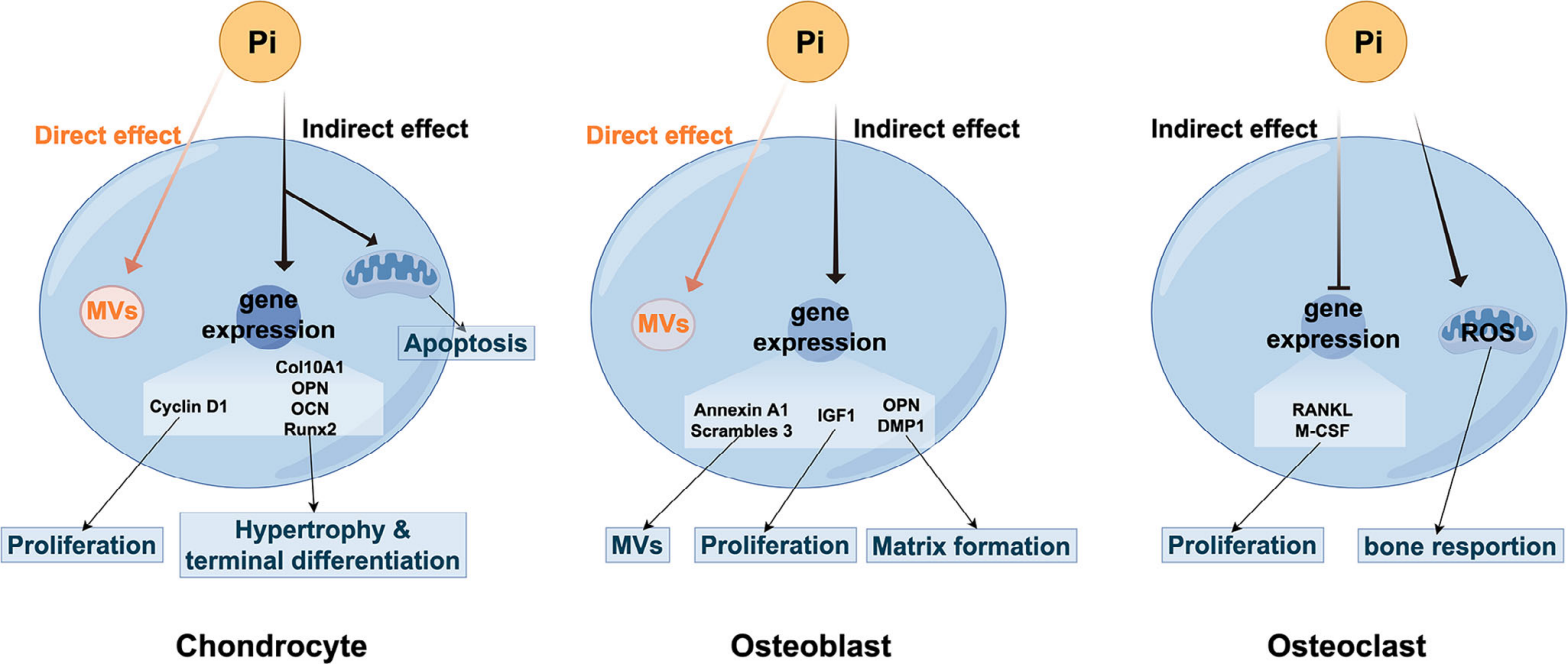
The regulatory effects of phosphate on the biological behaviors of bone‐related cells. In chondrocytes, Pi triggers the expression of genes related with proliferation, hypertrophy, and terminal differentiation. However, high levels of Pi induce chondrocyte apoptosis through the mitochondrial caspase‐3 pathway. In osteoblasts, Pi promotes the expression of OPN to increase matrix formation. It also stimulates the secretion of insulin‐like growth factor 1 (IGF1), which enhances cell proliferation in an autocrine fashion. Phosphate activates proton channels to induce the production of reactive oxygen species (ROS), which enhance osteoclast function and survival.

Apart from the direct regulation of biomineralization by phosphate balance maintained by mineralizing cells, phosphate also exerts a reverse effect on mineralizing cells to participate biomineralization process indirectly [117, 118]. Phosphate is important for signal transduction and cellular function in mineralizing cells. Moreover, it regulates the expression of genes related to proliferation, differentiation, and mineralization in mineralizing cells [119, 120, 121]. As a signaling molecule, phosphate can modulate the progression of mineralization based on its concentration, thus achieving precise control of biomineralization process (Figure 4) [122]. This regulatory function is integral to the feedback mechanisms within the biomineralization process.

#### 3.2.1 Effect of Phosphate on Chondrocytes

During endochondral skeletogenesis, the Pi concentration within extracellular environment of chondrocytes gradually increases. Pi exerts distinct role in chondrocytes proliferation, hypertrophy, terminal differentiation, and mineralization process [123, 124]. In proliferating chondrocytes, Pi upregulates cyclin D1 expression [125]. In the hypertrophic and terminal differentiation stages of chondrocytes, Pi induces the expression of PiT‐1, PiT‐2, and ankylosis (ANK), thereby creating a positive feedback loop to enhance Pi production and transport [126]. Phosphate also modulates the expression of hypertrophic and terminal differentiation marker genes, such as collagen type I alpha 1 (COLIA1), osteopontin (OPN) COL10A1, osteocalcin (OC), matrix metalloproteinase 13 (MMP‐13), and runt‐related transcription factor 2 (Runx‐2) which are essential for endochondral ossification [126, 127]. In mature cartilage, phosphate can upregulate matrix Gla protein, which is the inhibitor of mineralization [67]. This indicates the presence of a negative feedback loop for the control of mineralization processes within chondrocytes. When the concentration of phosphate is too high, it can lead to chondrocyte apoptosis [128]. Therefore, the alteration of Pi concentration can temporally modulate chondrocyte differentiation stages.

Conversely, PPi functions as a negative regulator of terminal differentiation and mineralization of chondrocytes [129]. It reduces the expression of hypertrophic and terminal differentiation markers, including ATPase, OC, and Runx‐2. This negative regulatory mechanism aims to prevent uncontrolled terminal differentiation and excessive mineralization [126]. Therefore, the PPi/Pi ratio is also crucial for mineralizing cells.

#### 3.2.2 Effect of Phosphate on Osteoblasts and Osteocytes

Pi plays distinct roles in osteoblast proliferation, differentiation, and mineralization process [130, 131, 132]. In the early stage, phosphate predominantly promotes the proliferation of preosteoblast cells. A mRNA microarray analysis of preosteoblast cells treated with Pi has identified multiple upregulated proteins related to cell cycle and proliferation [133]. During the mineralization stage, osteoblasts form a positive feedback phosphate regulatory system to regulate mineralization. The initial low levels of Pi stimulate the expression of ALP and PiT‐1, enhancing the Pi production and transport [134]. Increased Pi concentration upregulates the expression of proteins related to mineralization, including OPN, dentin matrix acidic phosphoprotein 1 (DMP1), and nuclearfactor erythroid‐derived 2‐like 2, which facilitates the mineralization process [135, 136, 137]. However, this regulatory mechanism is inactivated in the early osteoblastic differentiation, which prevents excessive mineralization before osteoblasts cease proliferation [138]. Recent research has elucidated that Pi can induce the production of MVs without transcriptional modifications, resulting in a faster response to Pi alteration [139]. Pi also induces the elevated expression of protein Annexin A1 and Scramblase 3 are closely related to MVs [140].

#### 3.2.3 Effect of Phosphate on Osteoclasts

Osteoclasts degrade bone tissues and are crucial in bone formation and remodeling [141]. In the resorption pit, the Pi concentration is elevated due to the degradation of CAP. Although the specific concentration is unknown, osteoclasts are exposed to high levels of Pi. Osteoclast differentiation, survival, and function can be promoted under suitable Pi concentration [142]. High or low levels of Pi can both inhibit the differentiation of osteoclasts [143, 144, 145]. The inhibitory effect of high Pi levels could be attributed to the suppression of RANKL and M‐CSF in a dose‐dependent manner [146]. High level of Pi also directly exerts an inhibitory effect on osteoclast precursor cells and induces the apoptosis of osteoclasts to dampen osteoclast's function [147].

### 3.3 The Indirect Effect: Protein Phosphorylation Mediates the Mineralization Process

Protein phosphorylation is the process in which specific amino acid residues in a protein molecule bind to phosphate groups, playing an important regulatory role in biological mineralization. In living organisms, calcium and phosphate are typically in a supersaturated state. Phosphorylation of proteins affects their interaction with minerals by altering charge density or protein conformation, thereby promoting or inhibiting the mineralization process [148, 149].

The degree of phosphorylation of proteins is crucial to their role in promoting or inhibiting mineralization. OPN is widely expressed in both soft and hard tissues, where it can adsorb and inhibit the growth of HAP crystals, playing a role in preventing mineral deposition in soft tissues and bodily fluids [150]. The regulatory effect of OPN on mineralization is related to its phosphorylation level. Dephosphorylated OPN loses its ability to inhibit mineralization, while highly phosphorylated OPN promotes the formation of HAP crystals. Phosphorylation of OPN alters its regulatory effect on mineralization, ensuring that mineralization occurs at the appropriate time and location [151].

Protein phosphorylation also supports the ordered formation of the structure of mineralized tissues. The monophosphorylation of Serine 16 in the enamel protein amelogenin enhances its ability to stabilize ACP, preventing the premature formation of HAP crystals. This inhibitory effect helps enamel rods grow in an ordered manner, forming a normal enamel structure [152]. In contrast, amelotin is expressed during the enamel maturation stage, and its conserved phosphorylation site, SSEEL, promotes the formation of HAP. Phosphorylated amelotin plays an important role in the formation of a dense enamel surface layer [153].

## 4 The Dual Function of Phosphate in Pathological Mineralization

The direct involvement of phosphate is widely recognized as an important factor in pathological mineralization, while the indirect role of phosphate has been reported in specific conditions such as vascular calcification, hyperphosphatemic familial tumoral calcinosis (HFTC) and hypophosphatemic rickets. Given the significant role of phosphate as a signaling molecule that regulates cellular activity, the indirect effect of phosphate in pathological mineralization should attract more attention in the future.

### 4.1 Vascular Calcification

Vascular calcification results from the pathological accumulation of calcium phosphate, primarily in blood vessels, valves, and the heart. The resulting ischemia in the heart or brain is a major contributing factor to patient mortality worldwide [154, 155, 156]. Vascular calcification is associated with aging, diseases that promote accelerated aging such as diabetes and chronic kidney disease (CKD), and specific genetic disorders related to ectopic calcification [157, 158, 159, 160, 161, 162]. These genetic disorders include pseudoxanthoma elasticum (PXE), generalized arterial calcification of infancy (GACI), and familial idiopathic basal ganglia calcification (IBGC) [154, 163, 164, 165, 166].

**FIGURE 5:**
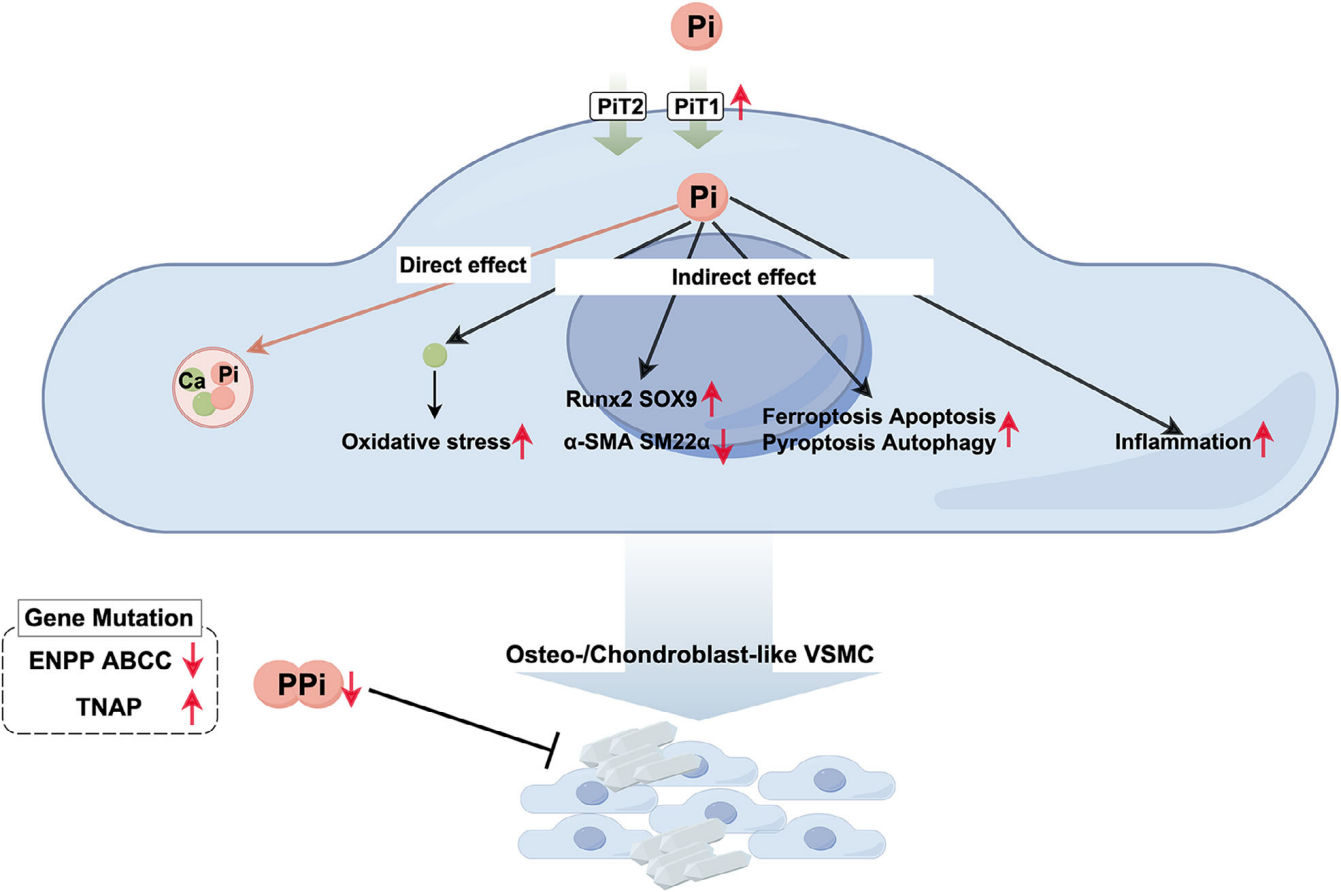
Alteration of Pi and PPi causing vascular calcification through direct and indirect effect. As for the direct effect, the elevated Pi levels promote vascular calcification, while the inhibitory effect on this process is downregulated due to the reduced PPi. As for the indirect effect, Pi can upregulate the osteogenic gene expression, enhance oxidative stress, trigger ferroptosis, apoptosis, pyroptosis and autophagy, and promote inflammation. Therefore, Pi can promote the transdifferentiation of vascular smooth muscle cell (VSMC) into osteo‐/chondroblast‐like VSMC and the subsequent vascular calcification.

Vascular calcification is initiated and progresses through the transdifferentiation of vascular smooth muscle cells (VSMCs) into osteo‐/chondrogenic‐like cells [167, 168, 169]. They can create a localized procalcifying environment enriched with calcium and phosphate and secrete MVs containing osteogenic proteins to promote mineralization [170]. The disruption of mineral homeostasis and elevated phosphate levels are recognized as key determinants for vascular calcification [171, 172]. Similar to the dual roles played in physiological mineralization, phosphate can directly and indirectly participate in the vascular calcification process (Figure 5) [173].

#### 4.1.1 The Direct Effect: Phosphate Imbalance in Vascular Calcification

Excess of Pi and reduction of PPi are considered as important causes of vascular calcification, directly impacting the calcification process [174]. High phosphate conditions have been extensively employed to induce the calcification of VSMCs as ex vivo model for vascular calcification investigation. In vitro investigations demonstrate that Pi aggravates VSMC calcification in a manner that is dependent on its concentration [175]. Particularly, high serum phosphate concentrations occur in diseases like CKD, which causes vascular calcification [176]. Additionally, in atherosclerosis plaques, abundant necrotic cells can release ATP at concentrations exceeding physiological levels. The subsequent metabolism of this ATP leads to the production of Pi rather than PPi, exacerbating the calcification of plaques [177]. The increase of Pi provides substantial substrates for vascular calcification.

Pyrophosphate deficiency is more frequently implicated in the development of vascular calcification. Under physiological conditions, extracellular PPi and other calcification inhibitors (matrix‐Gla proteins, and fetuin‐A) are effective in preventing vascular calcification by inhibiting CAP formation [178, 179]. In pathological states, the expression of these factors is diminished, with PPi being a key representative. The proteins involved in PPi synthesis, transportation, and hydrolysis are closely related to multiple genetic disorders featured by vascular calcification. Systemic ENPP1 deficiency may result in decreased PPi concentration, inducing calcification in patients with GACI [180]. Mutation of ATP‐binding cassette subfamily C member 6 (ABCC6) results in PXE, a disorder characterized by ectopic mineralization disorder and GACI, due to reduced plasma PPi levels [181, 182]. TNAP is activated by inflammatory cytokines and subsequently promotes PPi hydrolysis. This causes the transdifferentiation of VSMCs into chondrocyte‐like cells and subsequent endochondral ossification [183]. Overexpression of TNAP induces vascular calcification due to the reduction of tissue PPi [184]. In vitro studies also show that decreased ankylosis protein homolog (ANKH) expression is a direct target of Wnt in VSMC calcification [185]. The inhibitory impact of PPi on mineralization has been to prevent vascular calcification by restoring plasma PPi levels through increased ENPP1 expression [180] and ABCC6 expression [186], and inhibition of TNAP expression [187]. Direct oral administration of PPi helps increase circulating PPi concentration and prevent vascular calcification [181, 182].

Prior studies report a lack of correlation between circulating PPi and susceptibility to calcification. In animal models or patients with PXE, the decreased circulating PPi does not completely account for vascular calcification [187, 188, 189, 190, 191]. In Abcc6−/− mice overexpressing ENPP1, small mineralized foci still exist even with elevated PPi levels in plasma [190]. Inhibition of TNAP decreases VC in the Abcc6−/− mouse model without changing PPi levels [187]. Patients with reduced circulating PPi levels display completely different phenotypes [192]. Research suggests that the duration of exposure to low circulating PPi is a significant factor for VC and clinical severity in PXE [189]. Although PPi is an important mediator of ectopic calcification, plasma PPi may not serve as a reliable predictor for extracellular PPi in tissues with ectopic mineralization (Table 2).

**TABLE 2:** Involvement of phosphate alteration in the propagation of vascular calcification.

Phosphate metabolism process	Related gene	Clinical and experimental evidence	Is this supportive evidence that phosphate participates in pathological mineralization?	Refs.
Pi hydrolysis	NT5E, also known as CD73	Lack of CD73 results in increase in TNAP, a major enzyme in ectopic calcification.	Yes	[193]
Pi transport	SLC20A2/PiT‐2	In patients with IBGC caused by *SLC20A2* mutation, the Pi level in cerebrospinal fluid is significantly higher than that of the control.	Yes	[194, 195]
PPi synthesis	ENPP1	In GACI animal models, VC correlates with plasma PPi levels. An ENPP1‐Fc fusion protein may inhibit VC.	Yes	[180, 191]
		In GACI animal models, oral administration of PPi inhibits ectopic calcification.	Yes	[182]
	ABCC6 (involved in the efflux of ATP and subsequent generation of PPi)	Clinical evidence shows arterial calcification is weakly linked to circulating PPi levels in patients with pseudoxanthoma elasticum	No	[189]
		In *Abcc6^−/−^* mouse model, PPi concentration does not account for the therapeutic effect of TNAP inhibition on ectopic mineralization.	No	[187]
		In *Abcc6^−/−^* mouse model, overexpression of ENPP1 or administration of ENPP1‐Fc enzyme does not completely inhibit ectopic calcification despite increase in plasma PPi levels.	Unsure	[190, 191]
		In *Abcc6^−/−^* mouse model, oral administration of PPi inhibits ectopic calcification.	Yes	[181, 182]
		In *Abcc6^−/−^* mouse model, intravenous administration of ABCC6 increases PPi level in plasma and reduces ectopic mineralization.	Yes	[186]
PPi removal	TNAP	Mice with TNAP overexpression display generalized arterial calcification due to decreased tissue PPi.	Yes	[184]
		In cells from patients with arterial calcification caused by CD73 deficiency, TNAP activity increases as a compensatory response to the lack of CD73.	Yes	[196]
PPi efflux	ANKH	ANKH is the target gene of Wnt1 in the prevention of vascular calcification.	Yes	[185]

Abbreviations: ABCC6, ATP‐binding cassette subfamily C member 6; ANKH, ankylosis bone morphogenetic protein; CD73, cluster of differentiation 73; ENPP1, ectonucleotide triphosphate diphosphohydrolase 1; GACI, generalized arterial calcification of infancy; NT5E, ecto‐5′‐nucleotidase; PiT‐2, phosphate transporter 2; SLC20A2, solute carrier family 20 member 2; TNAP, tissue nonspecific alkaline phosphatase; VC, vascular calcification.

#### 4.1.2 The Indirect Effect: Phosphate Regulating Transdifferentiation of VSMCs

The elevated phosphate levels can induce VSMC transdifferentiation through complex signaling pathways, which exerts an indirect effect on the calcification process [197]. In VSMCs, the expression of PiT‐1 is significantly upregulated, leading to an increase in intracellular phosphate concentration [198]. The high phosphate concentration promotes the osteo‐/chondrogenic transdifferentiation of VSMCs and upregulates the expression of transcription factors muscle segment homeobox homolog of 2, Runx‐2, and SOX9, while simultaneously downregulating the expression of smooth muscle‐specific proteins such as α‐smooth muscle actin and smooth muscle protein 22‐α [173, 199]. Multiple pathways have been implicated in this process, including the downregulation of peroxisome proliferator‐activated receptor γ and activation of WNT/β‐catenin [200, 201].

Furthermore, high phosphate levels can induce VSMC calcification through the induction of ferroptosis, apoptosis, pyroptosis, and autophagy [202, 203, 204, 205, 206]. Studies have shown that high phosphate levels can downregulate the expression of SLC7A11, a cystine‐glutamate antiporter, and reduce the glutathione content in VSMCs. Additionally, the glutathione depletion induced by erastin, a small molecule initiating ferroptotic cell death, can significantly promote VSMC calcification [207]. High phosphate can induce the formation of apoptotic vesicles in a dose‐ and time‐dependent manner, which serves as a nidus for calcification [208, 209].

The inflammation of VSMCs induced by high phosphate levels plays an important role in the initiation and progression of ectopic mineralization [210, 211]. It promotes the release of inflammatory factors such as interleukin‐6, interleukin‐8, and tumor necrosis factor‐α [212]. And classic inflammatory pathways are also activated by high phosphate levels, including nuclear factor kappa‐B pathway and JAK‐STAT pathway [212, 213]. The activation of inflammation exacerbates the expression of osteogenic markers in VSMCs.

Additionally, calcium‐related signaling pathways are involved in the vascular calcification induced by high phosphate levels. It can activate depolarization‐triggered calcium influx via voltage‐gated calcium channels. The subsequent increase in intracellular calcium can induce oxidative stress and osteogenic differentiation [214]. High phosphate levels have also been shown to regulate the expression of calcium release‐activated calcium modulator 1, and stromal interaction molecule 1, thereby modulating store‐operated calcium entry in VSMCs [199]. The specific mechanisms by which phosphate influences these pathways are an active area of investigation.

There is a negative feedback protective mechanism against phosphate‐induced vascular calcification. High phosphate levels elevate the expression of FGF23, which can bind to Klotho to inhibit vascular calcification [215, 216, 217]. Simultaneously, FGF23, as the main regulator of serum phosphate, can lower serum phosphate levels when elevated [218].

### 4.2 Nephrocalcinosis

Nephrocalcinosis is defined by the generalized accumulation of calcium oxalate or calcium phosphate in the kidney parenchyma. Various genetic disorders associated with metabolic abnormalities promote the development and progression of nephrocalcinosis [219, 220, 221]. Phosphate metabolism disorders contribute to nephrocalcinosis by directly causing the deposition of calcium phosphate in the kidneys, or by triggering hypercalciuria through endocrine pathways [222, 223, 224].

High phosphate intake can lead to hyperphosphatemia and hyperphosphaturia, resulting in acute nephrocalcinosis. This phenomenon was first observed in some patients who developed extensive renal tubular calcification after taking oral sodium phosphate before a colonoscopy. The supersaturated calcium and phosphate in the kidneys are deposited in the form of calcium phosphate crystals, which exert a direct role in this ectopic mineralization process [225, 226]. Tamm–Horsfall protein (THP) and OPN play a regulatory role in nephrocalcinosis caused by mineral salt deposition. Under normal conditions, OPN and THP inhibit the deposition of supersaturated mineral salts in the kidneys. The absence of OPN and THP can lead to the formation of calcium phosphate crystals. Once calcium phosphate crystals have formed, other types of urinary crystals can also promote crystal growth, eventually leading to significant mineral deposition in the kidneys [227, 228, 229].

Hypophosphatemia causes the downregulation of FGF23 and upregulation of 1,25(OH)_2_D_3_. Elevated levels of 1,25(OH)₂D₃ enhance intestinal calcium absorption, leading to absorptive hypercalciuria. Hypophosphatemia may cause or exacerbate hypercalciuria through an endocrine pathway, thereby affecting nephrocalcinosis. Hypercalciuria, is a predominant pathological feature of nephrocalcinosis. Mutations in genes related to calcium metabolism, such as *CLCN5*, *CYP24A1*, *SLC34A1*, *CLDN16*, *CLDN19*, and *CASR*, have been reported to induce hypercalciuria and subsequently nephrocalcinosis. These mutations are also known to disrupt phosphate metabolism [216]. For example, the mutation of *CLCN5* alters the expression of the sodium/proton exchanger NHE3, which in turn affects sodium reabsorption. This change indirectly impairs phosphate reabsorption, leading to hypophosphatemia [230]. Similarly, NaPi‐IIa is the major regulator of renal phosphate reabsorption and mutations in the SLC34A1 gene disrupt NaPi‐IIa function, leading to hypophosphatemia [231]. The increase in calcium and phosphate supersaturation exacerbates the risk of calcium phosphate deposition in the renal tubules, leading to nephrocalcinosis. Consequently, hypercalciuria caused by hypophosphatemia may be the primary reason for nephrocalcinosis [232].

### 4.3 Corneal Calcification

Corneal calcification is an ectopic mineralization that occurs in the superficial layers of the cornea, typically resulting from abnormal serum calcium and phosphate metabolism [233, 234]. This condition often manifests as calcific band keratopathy, characterized by the accumulation of calcium phosphate in the Bowman's layer, the basement membrane of the corneal epithelium, and the most superficial anterior corneal stroma [235]. Elevated phosphate levels have been shown to contribute to the development of corneal calcification directly. Additionally, phosphate also acts as a signaling molecule to regulate calcification of corneal epithelial cells.

Calcium and phosphate in tears exist in a delicate equilibrium. Disturbances in this equilibrium can induce calcium phosphate precipitation. The use of phosphate‐containing ophthalmic drugs has been associated with corneal mineralization [236]. Studies have reported that crystalline calcium phosphate deposits form with the application of phosphate‐buffered hyaluronate artificial tears, which have a phosphate concentration (50.9 mmol/L) that is 50 times higher than that of normal serum [237]. Using phosphate‐buffer saline for eye rinsing during the treatment of ocular burns also induces corneal mineralization [238]. The precipitation of extracellular calcium phosphate appears to result from local events. These events include increases in the concentration of calcium or phosphate in the immediate area [235].

Recent studies have confirmed that phosphate and calcium act synergistically as signaling molecules to regulate corneal epithelial cells. An excess of phosphate and calcium upregulates RUNX2 protein expression in corneal epithelial cells in a dose‐dependent manner and induces its nuclear translocation, leading to the calcification of corneal epithelial cells [239].

### 4.4 Hyperphosphatemic Familial Tumoral Calcinosis

HFTC is a rare autosomal recessive metabolic disorder characterized by the accumulation of calcium phosphate and CAP in the periarticular soft tissue, resembling a neoplasm [240, 241]. Patients with HFTC exhibit hyperphosphatemia with normocalcemia. This finding is indicative of the major involvement of phosphate in ectopic mineralization [242, 243]. The hyperphosphatemia is due to increased renal phosphate reabsorption, which is influenced by decreased activity of FGF23. This disease results from mutations in *FGF23* and its regulators, including the *GALNT3* and *KLOTHO* genes [242, 244, 245]. Calcium phosphate precipitates due to hyperphosphatemia usually develop in areas of inflammation, tissue hypoxia, or repetitive trauma, although the exact etiology remains unclear [246]. Conservative treatment for HFTC aims to lower serum phosphate concentration through a phosphate‐restricted diet, noncalcium‐based phosphate binder tablets (sevelamer hydrochloride), and oral acetazolamide to induce phosphaturia [242, 247]. In patients with severe hyperphosphatemia, hemodialysis may be employed as an extreme measure to decrease phosphate levels [248].

The indirect role of phosphate in the accumulation of calcium phosphate and CAP in HFTC has been reported [249, 250, 251]. Hyperphosphatemia has been shown to induce the expression of FGF7 in fibroblasts, which is exclusively produced in the dermal layer. This could explain the predominant occurrence of ectopic calcification in cutaneous and subcutaneous tissues [249, 250]. Additionally, the activation of MMPs has been implicated in this process, which has been shown to mediate ectopic calcification in vascular tissues [250]. Although the investigation on the direct role of phosphate is limited, it is reasonable to infer that the elevated levels of phosphates in hyperphosphatemia provide substantial substrates for mineral deposition.

### 4.5 Breast Microcalcifications

Breast microcalcifications are calcium salt deposits within breast tissue, considered suspicious signs of breast cancer, and are associated with a poorer prognosis in breast cancer. At the molecular level, they are classified into Type I, composed of calcium oxalate, and Type II, composed of CAP [252, 253, 254]. High phosphate levels are one of the characteristics of the breast cancer microenvironment. This results from the phosphate accumulation capacity of breast cancer cells. Compared to normal cells, breast cancer cells overexpress the NaPi‐IIb transporter and H^+^‐dependent phosphate transport proteins to absorb additional Pi [140, 255].

The elevated phosphate could serve as a substrate for breast microcalcifications directly. A proposed mechanism for breast microcalcification suggests that ALP on the surface of mammary cells hydrolyzes organic phosphate to produce Pi. This Pi is subsequently transported into cells and combines with calcium ions to synthesize CAP crystals [256]. Moreover, ALP reduces the inhibitory effect of OPN on mineralization. This is achieved by dephosphorylation and hydrolysis of PPi to Pi, thereby eliminating the inhibitory effect of PPi on CAP crystal growth [256, 257, 258].

In the calcified regions surrounding breast cancer cells, cells with osteoblast‐like characteristics have been identified. This may result from the epithelial–mesenchymal transition of breast epithelial cells under certain stimuli, and it could be related to the formation of microcalcifications [259]. Whether phosphate promotes the occurrence of cells with osteoblast‐like characteristics needs further investigation.

### 4.6 Calcium Pyrophosphate Deposition Disease

This disease is also known as chondrocalcinosis articularis and is caused by calcium pyrophosphate (CPP) crystals. It includes conditions such as acute CPP crystal arthritis, chronic CPP crystal inflammatory arthritis, and osteoarthritis with calcium pyrophosphate deposition (CPPD) [260, 261, 262]. Acute manifestations of the disease include warmth and swelling in and around the joint due to the severe inflammatory response to CPP crystals. The chronic manifestation is a polyarticular form of arthritis similar to osteoarthritis [263]. While the exact pathogenesis of CPPD disease remains unclear, the deposition of CPP crystals in the pericellular matrix of cartilage is undoubtedly the predominant etiological factor. The CPP crystals cause tissue damage by initiating inflammation, through activation of NLRP3 inflammasomes. They also impose catabolic effects on chondrocytes by inducing the production of destructive matrix metalloproteinases [263, 264, 265, 266, 267].

The low Pi/PPi ratio is the major determinant for CPP crystal formation, reflecting an abnormal direct involvement of phosphate in mineralization. The high level of extracellular PPi in synovial fluid is produced from the pyrophosphohydrolysis of nucleoside triphosphates (NTP) by nucleotide pyrophosphohydrolase (NTPPH) enzymes and is transported by ANK [263, 268]. CPP crystals form in the pericellular cartilage matrix or articular cartilage vesicles, which contain NTPPH enzymes and TNAP enzymes. Chondrocytes secrete more NTP under mechanical stress, which, in turn, elevates PPi concentration. Excess extracellular PPi then forms CPP crystals with calcium [268]. Mutation of *ANKH* has been shown to increase PPi transport function, resulting in elevated PPi concentration and familial CPPD [263, 268]. Future investigations should focus on identifying drugs that can lower PPi concentration and prevent the cartilage changes associated with CPPD [268, 269, 270, 271].

### 4.7 Hypophosphatemic Rickets

In addition to triggering ectopic calcification in abnormal locations, phosphate imbalance can also disrupt or impair the mineralization process in normal sites, leading to a range of mineralization deficiency‐related diseases that compromise bone strength and stability.

Hypophosphatemic rickets, a skeletal disorder caused by hypophosphatemia, is the most common disturbances of phosphate homeostasis [272, 273]. This condition includes several forms: autosomal dominant hypophosphatemic rickets (ADHR) caused by *FGF23* mutations, autosomal recessive hypophosphatemic rickets (ARHR) caused by *DMP1* mutations, and X‐linked hypophosphatemic rickets (XLH) caused by *PHEX* mutations [274, 275, 276, 277]. Hypophosphatemic rickets is featured by renal phosphate wasting, hypophosphatemia, and osteomalacia. Hypophosphatemia results in mineralization defects in the growth plates, bone, and teeth [278]. The bone tissue around osteocytes remains unmineralized, forming periosteocytic lesions [279].

In these diseases, altered FGF23 in osteocytes decreases serum phosphate and causes hypophosphatemia [280]. In ADHR, *FGF23* mutation prevents degradation of the altered protein. In ARHR, reduced DMP1 increases FGF23 expression. In XLH, *PHEX* mutation decreases 7B2 protein expression [281, 282]. This reduces 7B2·PC2 enzyme activity and suppresses FGF23 degradation. Elevated FGF23 causes renal phosphate wasting and hypophosphatemia [278]. Inactivating FGF23 antibodies helps maintain the stability of serum phosphate. This clinical treatment strategy highlights the participation of FGF23 in the pathogenesis of hypophosphatemic rickets [279].

There is no doubt that the reduction of phosphate directly affects the deposition of CAP in bone due to the decreased availability of substrates. In physiological mineralization, the process commences 2 weeks after osteoblasts secrete bone matrix. This delay is called mineralization lag time. Insufficient phosphate extends this lag time, resulting in the accumulation of osteoid (unmineralized bone matrix) [279]. Furthermore, the elevated secretion of FGF23 by osteocytes triggers the accumulation of PPi through autocrine and paracrine suppression of TNAP, which exerts an inhibitory effect on mineralization [283].

Regarding the indirect role of phosphate in hypophosphatemic rickets, chondrocytes, osteocytes, and osteoclasts are all involved in this process. Low phosphate levels accelerate chondrogenesis and promote the transdifferentiation of chondrocytes to bone cells [284]. Additionally, it impairs the caspase‐mediated apoptosis of hypertrophic chondrocytes [285]. Furthermore, low phosphate levels can lead to a reduction in the number of osteocytes due to increased osteocyte apoptosis, which also hinders osteocyte maturation and osteoid accumulation [286]. The impact of low phosphate levels on osteocytes is also associated with the pathogenesis of skeletal abnormalities in hypophosphatemic rickets. It results in a decreased osteoclast number and inhibition of osteoclast differentiation and function [287].

## 5 Therapeutic and Translational Applications

The treatment of phosphate metabolism disorders needs to be based on pathological mechanisms and disease specificity. Bisphosphonates and phosphate binders are the common clinical drugs for osteoporosis and hyperphosphemia, respectively. For hypophosphatemia, traditional phosphate supplements require frequent administration and may induce secondary hyperparathyroidism. To address these issues, emerging technologies such as nanodelivery systems have been developed for phosphate supplements. Additionally, targeting phosphate transporters has become a research hotspot. Specific molecules are also involved in different diseases related with phosphate imbalance‐induced pathological mineralization.

### 5.1 Bisphosphonates and Phosphate Binders

Bisphosphonates are widely used as first‐line treatments for impaired bone metabolism, such as osteoporosis, Paget's disease, osteogenesis imperfecta, and metastatic bone cancer [288, 289]. Structurally similar to PPi, they bind to HAP via a P–C–P central group and are absorbed by osteoclasts. Once internalized, bisphosphonates dissociate from HAP and inhibit osteoclast activity through various mechanisms. The first generation, non–nitrogen‐containing bisphosphonates (non‐NBPs), interacts with aminoacyl‐tRNA synthetase to form nonhydrolyzable ATP analogs, inducing apoptosis. In contrast, nitrogen‐containing bisphosphonates (second and third generations) inhibit the mevalonate pathway, exhibiting a stronger proapoptotic effect on osteoclasts [290, 291]. However, long‐term use has been associated with osteonecrosis of the jaw and atypical femoral fractures [292, 293].

Phosphate binders are mainly used for the treatment of CKD mineral bone disorder, especially hyperphosphatemia [294, 295]. Phosphate binders reduce phosphate absorption by combining with dietary phosphate in the gastrointestinal tract to form insoluble complexes. These insoluble complexes are subsequently excreted through feces, reducing serum phosphorus levels. Different phosphate binders combine with negatively charged phosphate through different cations to form insoluble complexes [296]. Although phosphate binders are effective in reducing serum phosphorus levels, they also have some limitations. For instance, calcium‐based phosphate binders may lead to hypercalcemia and vascular calcification, while noncalcium‐based phosphate binders such as selveram may cause gastrointestinal discomfort [297, 298].

### 5.2 Novel Phosphate Delivery Systems

Phosphate supplements represent another commonly used kind of phosphate medications, generally in the form of sodium or potassium salts. Hypophosphatemia, resulting from renal phosphate wasting, often leads to diseases such as rickets, progressive growth failure, and osteomalacia due to phosphate deficiency [299]. The conventional treatment involves oral administration of phosphate to temporarily elevate phosphate levels. After oral phosphate supplementation, serum phosphate levels increase rapidly. However, due to the relatively short half‐life of oral phosphate supplements, long‐term and frequent administration is required [300]. Furthermore, it is imperative to consider the potential complications associated with the excessive utilization of phosphate, such as secondary or tertiary hyperparathyroidism. Concurrent use of active vitamin D can reduce the occurrence of this issue [301]. Additionally, it is necessary to monitor urine calcium levels to prevent the occurrence of nephrocalcinosis [302].

A variety of new phosphate delivery systems are emerging and showing great potential in the biomedical field. Among them, calcium phosphate nanoparticles are regarded as highly promising drug delivery materials due to their excellent biocompatibility, biodegradability, and strong affinity for nucleic acids (such as pDNA, siRNA, miRNA) and various therapeutic drugs (such as cisplatin, carboplatin, paclitaxel, etc.) [303]. Researchers have developed a variety of strategies aimed at preparing calcium phosphate nanocarriers with controllable size, good stability, targeting ability, and pH sensitivity, making them potential clinical candidates [304]. Nanoparticles coated with cell membranes are another type of important delivery system. Such nanoparticles ingeniously utilize the natural properties of cell membranes, such as the ability to target specific tissues and immune escape mechanisms, thereby significantly enhancing the efficiency of drug delivery. Phosphate–phosphonate hybrid nanomaterials represent a unique delivery strategy. Unlike traditional electrostatic adsorption or organic covalent bonding methods, this type of material uses surface grafting chemical methods to prepare oligonucleotide arrays. This method can provide a stable and clear interface, in which oligonucleotide probes with phosphate groups at the ends are directly attached to the zirconium surface to form covalent connections [305].

### 5.3 Strategies Targeting Phosphate Transporters

Apart from phosphate supplements and phosphate binders, modulating phosphate transport is another effective way to treat phosphate imbalance‐related diseases. Multiple drugs have been reported to inhibit phosphate transport, such as phosphonoformate, phosphorylated 2′‐phosphophloretin, nicotinamide adenine dinucleotide, arsenate, and triazole derivatives [306]. However, the questions about their ineffectiveness and toxicity need to be addressed.

Current research is focused on developing more effective and safer drugs. Tenapanor has been approved by FDA in 2023 to treat hyperphosphatemia in patients with CKDs. It reduces serum phosphate levels through reducing the paracellular phosphate absorption and the expression of NaPi‐IIb [307]. Several other promising drugs have entered clinical trials. The novel pan‐phosphate transporter inhibitor EOS789 can markedly reduce the serum phosphate level, through the interaction with NaPi‐IIb, PiT‐1, and PiT‐2. The application of EOS789 has been reported to alleviate ectopic calcification of the thoracic aorta [324] and kidney injury in antiglomerular basement membrane nephritis [308, 309]. The efficacy and safety of EOS789 have been proved in a phase 1b randomized crossover trial in hemodialysis patients [310]. AP306, another pan‐inhibitor of phosphate transporter, also shows great potential in reducing serum phosphate levels in patients receiving hemodialysis with hyperphosphatemia. The efficacy and safety of AP306 have been demonstrated through a phase 2 clinical trial [311]. Additionally, some novel molecules exhibit potential as phosphate transport inhibitors in preclinical animal experiments. PF‐06869206, a NPT2a‐selective small‐molecule inhibitor, can also reduce serum phosphate level in rats with CKD [312, 313, 314]. LY3358966 is a NPT2b inhibitor that can reduce serum phosphate levels [315].

### 5.4 Disease‐Specific Treatment Strategies

#### 5.4.1 Vascular Calcification

At present, the treatment strategies for vascular calcification mainly include drug therapy, interventional therapy, and the application of new nanomedicines. In terms of drug treatment, phosphate binders and calcium simulants have been proven to slow down the progression of vascular calcification in patients with CKD, while vitamin K supplements, pyrophosphates, and so forth are also being studied, showing certain potential [316].

Furthermore, sodium thiosulfate, as a clinically approved antivascular calcification drug, has limited efficacy due to its low bioavailability and severe side effects. To address this issue, researchers have developed a biomimetic nanocarrier based on grapefruit‐derived extracellular vesicles for the safe and targeted delivery of sodium thiosulfate. This nanomedicine has demonstrated excellent cellular uptake ability, inhibitory effect on the calcification of VSMCs, without obvious systemic toxicity [317]. Furthermore, the research also found that the natural product Moscatilin (moscatilin) can inhibit vascular calcification by activating IL13Ra2‐dependent STAT3 to inhibit the WNT3/β‐catenin signaling pathway [318]. Future research directions may include a more in‐depth exploration of the genetic basis of vascular calcification, and the development of new imaging techniques that can more precisely distinguish intimal and media calcification to achieve more precise treatment.

#### 5.4.2 Nephrocalcinosis

Sulforaphane, a Nrf2 activator, significantly inhibits the inflammatory response and renal tubular epithelial cell damage caused by calcium oxalate renal calcification. It promotes the transcription of miR‐93‐5p by upregulating Nrf2, thereby targeting and inhibiting the expression of TLR4 and IRF1, alleviating the inflammatory response, and thus reducing the deposition of calcium oxalate crystals [319]. Additionally, OPN has a significant protective effect on renal calcification under high phosphate load. OPN may affect phosphate metabolism by regulating the expression of FGF‐23, thereby alleviating renal calcification [320]. Furthermore, inhibiting the activation of the NLRP3 inflammasome can significantly alleviate CKD related to renal calcification [321]. Finally, for renal calcification caused by mutations in the SLC34A1 and SLC34A3 genes, although phosphate therapy improved biochemical indicators to a certain extent, the effect was limited [322].

#### 5.4.3 Corneal Calcification

For mild corneal calcification, the local application of edetic acid chelating agent can effectively remove calcium deposits on the corneal surface [323]. Lamellar keratoplasty can be used in cases where corneal calcification involves deeper tissues. Corneal function is restored by removing the diseased tissue and replacing it with healthy corneal tissue [324]. For calcification caused by corneal neovascularization, photodynamic therapy combined with subconjunctival injection of bevacizumab may be an effective treatment option [325]. It is worth noting that for corneal calcification caused by systemic diseases such as chronic renal failure and hyperparathyroidism, controlling the underlying systemic diseases is the key to treatment.

#### 5.4.4 Hyperphosphatemic Familial Tumoral Calcinosis

Traditional treatment methods of HFTC include restricting phosphate intake, using phosphate binders and/or administering carbonic anhydrase inhibitor acetazolamide to reduce the reabsorption of phosphorus by the kidneys [326]. Burosumab is a monoclonal antibody against FGF23. By binding to and inhibiting the activity of FGF23, it increases the excretion of phosphorus by the kidneys, thereby reducing blood phosphorus levels [327]. Surgical removal is performed for obvious symptoms caused by the mass, such as pain and functional limitations [328].

#### 5.4.5 Breast Microcalcifications

Breast microcalcification is an important biomarker for the diagnosis of breast cancer, and the choice of treatment strategy depends on the pathological results of microcalcification [329]. For benign microcalcification, regular follow‐up is usually only required. For malignant microcalcification, individualized treatment plans need to be formulated based on factors such as the type and stage of the tumor, the patient's age and overall health status. Common treatment methods include surgical resection, combined radiotherapy, chemotherapy, endocrine therapy, and targeted therapy [330, 331]. During the treatment process, imaging re‐examination is crucial for evaluating the treatment effect and monitoring recurrence.

#### 5.4.6 Calcium Pyrophosphate Deposition Disease

The treatment strategies for CPPD mainly focus on controlling inflammation and alleviating symptoms, as there are currently no drugs capable of dissolving the CPP crystals that cause the disease. During the acute attack period, prednisone is the preferred drug because it shows good efficacy in relieving pain and inflammation. Colchicine is also an effective option, especially in low‐dose regimens, but it may cause mild diarrhea and should be used with caution especially in elderly patients. For refractory cases, biological agents such as IL‐1 inhibitors (such as anabelidin) and IL‐6 inhibitors (such as tocilizumab) have shown certain efficacy. Surgical treatment is usually used for patients with single‐joint involvement [332, 333, 334].

#### 5.4.7 Hypophosphatemic Rickets

The treatment of XLH mainly relied on oral phosphate supplements and active vitamin D analogues (such as calcitriol or α‐calcitriol). Phosphate supplements are used to correct hypophosphatemia, while active vitamin D is used to enhance the intestinal absorption of calcium and phosphate [335]. However, careful monitoring is required to avoid hypercalcemia and hypercalciuria. Recently, Burosumab, an anti‐FGF23 monoclonal antibody, has brought new hope for the treatment of XLH. The main cause of XLH is the excessive expression of FGF23, which leads to a reduction in the reabsorption of phosphate in the kidneys and subsequently causes hypophosphatemia. Burosumab increases serum phosphate levels and improves bone mineralization by blocking the action of FGF23 and reducing phosphate loss. Clinical trials have shown that compared with traditional treatments, Burosumab is more effective in improving rickets, bone deformities, height and bone mineralization. However, extra caution is needed when using Burosumab in CKD patients, as it may increase the risk of hyperphosphatemia [336, 337, 338].

## 6 Research Gaps and Perspectives

### 6.1 Technical Limitations

Although phosphate is essential for these biological processes, the mechanisms of phosphate sensing, storage, and release remain inadequately understood in osteoblasts and other cells, especially when compared with calcium. Several factors contribute to this knowledge gap. First, phosphate‐involved reactions within biological systems are considerably more intricate than those involving calcium. This complexity arises from the fact that phosphate exists in various forms within cells, including organic phosphate (phospholipids and phosphate esters) and inorganic phosphate (PO_4_^3−^ ion/Pi, PPi), whereas calcium primarily exists in ionic form [339, 340, 341]. Second, the function of phosphate is more complex than calcium within cells. Apart from its role as mineral substrate and signaling molecules, similar to calcium, phosphate is also an essential component of cellular structures, including cell membranes and ATP. Furthermore, there is a lack of effective visual and quantitative detection methods for cellular phosphate due to the diverse forms of phosphate existence. In contrast, calcium can be converted into fluorescent signals using calcium‐sensitive indicators such as Fura‐2 acetoxymethyl ester [342, 343]. And single‐cell calcium quantification can be performed through inductively coupled plasma optical emission spectrometer [344, 345]. However, the detection of phosphate is complicated through this method due to the existence of organic phosphate and inorganic phosphate within cells.

### 6.2 Role of Microbiome in Phosphate Metabolism

Apart from the physiological and pathological mineralization in human, phosphate‐related mineralization also occurs in microbes. These microbes secrete phosphatase, phytase, or acids to solubilize the phosphorus sources and generate inorganic phosphate, leading to the precipitate heavy metal‐phosphate minerals. This process is known as microbial‐induced phosphate precipitation [346]. Heavy metals (such as Cd, Cu, Pb, Zn, and U) could be transformed into stable and harmless phosphate minerals by bacteria with phosphate‐solubilizing and the pH‐regulating abilities [347, 348]. Therefore, phosphate‐mineralizing bacteria have been widely applied for heavy metal contamination remediation [349]. For these bacteria, phosphorus metabolism genes such as pst, pit, phn, ugp, ppk, and so forth are of great importance [350]. The involvement of bacteria makes it possible for the removal of heavy metals contamination with low concentration. Even when uranium concentrations are < 1 µM, *Caulobacter* sp. (strain OR37) could induce the formation of uranium‐phosphate mineral to lower the uranium concentrations [351].

As for the medical application of biomineralized bacteria, the most common strategy is enveloping bacteria in a mineralized coating to enhance antitumor immunotherapy [352]. For example, the administration of mineralized *Salmonella typhimurium* could suppress the growth of tumors [353]. Additionally, the coating of calcium phosphate could reduce the systemic toxicity of bacterial membrane vesicles [354]. However, there are no reports about the bacteria‐induced mineralization in medical application. The reason may be the uncontrollability of bacterial mineralization in vivo. In the future, the involvement of genetic editing technologies might pave the way for controllable bacteria‐induced mineralization and realize the application for mineralization‐related diseases treatment.

### 6.3 Future Directions for Precision Mineralization Modulation

While the current review has summarized the importance of phosphate in both physiological and pathological mineralization, several gaps in our understanding remain. Future research should aim to reduce these gaps and realize the precision mineralization modulation. A significant gap that hinders the design of advanced treatment strategies is the incomplete understanding of the mechanisms of phosphate sensing and regulation. Recent studies have highlighted the role of PiT‐1 and PiT‐2 in detecting extracellular phosphate levels [64, 355, 356]. However, the precise mechanisms by which these transporters influence intracellular signaling pathways remain unclear. Future research should focus on identifying other potential phosphate sensors and elucidating the molecular mechanisms underlying phosphate sensing and regulation. In this context, the discovery of PXo bodies, a new organelle involved in regulating cellular phosphate homeostasis, opens up exciting possibilities. The PXo‐Cka‐JNK signaling cascade has been implicated in controlling tissue homeostasis through Pi‐dependent mechanisms. Investigating the role of PXo bodies in both physiological and pathological mineralization processes may provide new insights into phosphate metabolism and its regulation [357].

Another area of interest is the interorgan communication in phosphate homeostasis. This homeostasis is maintained through complex interactions between the intestines, kidneys, bones, and parathyroid glands [49, 358, 359]. While the roles of individual organs have been extensively studied, the mechanisms of interorgan communication remain less well understood. Future research should plan to elucidate how signals are transmitted between organs, and how these interactions contribute to maintaining phosphate balance. Understanding these mechanisms may generate new therapeutic strategies for disorders of phosphate metabolism.

Because genetic mutations have been implicated in abnormal mineralization resulting from phosphate imbalance, such as hypophosphatemic rickets and HFTC, future research should focus on identifying additional genetic factors that contribute to these conditions, and understanding how these mutations disrupt normal phosphate homeostasis [360, 361]. Advanced genomic and proteomic techniques should be used to provide deeper insights into the molecular basis of these disorders. This may result in the development of personalized treatment modalities.

Contemporary treatment strategies for phosphate metabolism disorders, such as the use of phosphate binders and inactivating FGF23 antibodies, have shown promise [362]. However, there is a need for more effective and targeted therapies. Future research should explore novel therapeutic agents that can modulate phosphate levels more precisely. Investigating the potential of gene therapy, small‐molecule inhibitors, and other advanced treatment schemes should expand the potential for better management of phosphate‐related mineralization disorders.

The identification of reliable biomarkers for phosphate‐related mineralization disorders is essential for early diagnosis and effective treatment. Future research should aim to develop and validate biomarkers that can accurately reflect phosphate homeostasis and predict disease progression. Advanced imaging techniques, coupled with molecular and biochemical assays, may enhance our ability to diagnose and monitor phosphate‐related mineralization disorders.

## 7 Conclusions

Phosphate plays an indispensable role in physiological and pathological mineralization processes. In physiological contexts, phosphate functions as a building block for mineralization, and as a signaling molecule that controls the differentiation, proliferation, and function of mineralizing cells. This multifaceted role illustrates the complexity of phosphate homeostasis, which is maintained through complex regulatory networks involving sensing, transport, storage, and excretion processes. Disturbances in these processes may result in various pathological conditions, including ectopic mineralization in soft tissues and abnormal mineralization in hard tissues. While significant progress has been made in understanding the role of phosphate in physiological and pathological mineralization, much remains to be discovered. By advancing our knowledge in these areas, we can improve the prevention, diagnosis, and treatment of phosphate‐related mineralization disorders to enhance patient outcomes and their quality of life.

## Author Contributions

Wen Qin, San‐yang Yu, and Jia‐lu Gao performed literature searching, made graphics, and wrote the manuscript. Jian‐fei Yan and Qian‐qian Wan organized the tables. Shuai‐lin Jia, Franklin Tay, and Kai Jiao researched the related references and participated in discussion. Lina Niu designed and edited the manuscript. All the authors have read and approved the manuscript.

## Ethics Statement

The authors have nothing to report.

## Conflicts of Interest

The authors declare no conflicts of interest.

## Data Availability

The authors have nothing to report.
